# Extracts from Clinique of the College of Physicians and Surgeons

**Published:** 1847-01

**Authors:** 


*Extracts from Clinique of the College of Physicians and Surgeons, by Prof. W. Parker, N. Y.; reported for the N. Y. Annalist.*

*Simple Fracture of both Bones of the Leg.*—Boy, æt. 12 years. A week ago yesterday, fell down a cellar and injured his leg; has been unable to walk since; has not been seen by any physician. In the first place, on looking at the limb and observing its color, we know that there has been a contusion, with extravasation of blood, and that it has reached the second stage. In the first stage, the discoloration is black ; after a time the red coloring matter of the blood is absorbed, and the limb assumes a yellow-ochre color, marking the second stage ; it then grows paler and disappears.— But what else are we to look for? The simple contusion will not account for the inability of motion. You observe that the patient supports the foot, and on withdrawing the support and allowing the foot to hang, he complains of pain. This well marked symptom would lead me to suspect fracture.

In determining then whether we have a fracture or not, we have the symptoms as gathered from the patient, and those resulting from manual examination ; if fracture of the femur, deformity is generally a very prominent one, but here this symptom is absent, which fact, to you, enhances the interest of the case. In the next place, you hear the patient complain of pain, and lastly of inability of motion. On manual examination, I get a false point of motion and crepitus; but it is not the *osseous* crepitus, and why? Nine days have elapsed since the accident, and an exudation of a viscid, gelatinous matter has taken place from the broken surfaces; a rudimentary callus is already partially developed. The interposition of this substance would of course prevent osseous crepitation, but furnishes another variety not less important in this matter of fractures; viz., *albuminous* crepitus. Usually in these cases, the leg is bowed, and from the superior power of the extensor over the flexor muscles the convexity of the curve always looks forward; but here the displacement is scarcely discoverable. On elevating the outer margin of the foot, the patient complains of pain at the point of fracture.

Both the tibia and fibula are broken in this case, at the junction of the lower with the middle third; the usual seat of fracture when both bones are broken. And here I would remark, that in children of this age, the direction of fracture of the extremities, is neither oblique, as in adults, nor transverse, as in old age, but the bone breaks as a hickory stick would, and this perhaps will account for the want of displacement in this case.

As to the treatment, let me say that permanent extension and counter extension are unnecessary, and liable to do far more harm than good. I do not mean to say that they must always be dispensed with, but I do say that the tight dressings, splints, and apparatus for extention and counter extension, with the necessary confinement, will, in these children, sometimes produce a vast amount of harm. Indeed I have known lives sacrificed, by the adoption of these means to prevent a little shortening of the limb. If you do get shortening of a quarter of an inch or so, it is easily remedied by the shoe-maker.

My plan is simply this; in the first place, I apply a roller, to render the pressure of the splint more equable. Then a splint made of book-binder's board, rendered soft and moist by soaking in water, and coated with a strong solution of glue or starch, is nicely adjusted to the limb, and retained in its place by a roller firmly applied. To facilitate its drying, warm bricks, or the ordinary domestic iron may be made use of; and your limb is then set. I omitted to state that the semiflexed position is the position in fractures of this kind; this position, with slight extension of the foot, freely relaxes the powerful extensor muscles of the foot. The child is not to be confined, but may be allowed to mingle in its accustomed gambols as before.

